# Mortality risk and temporal patterns of atrial fibrillation in the nationwide registry

**DOI:** 10.1002/joa3.12643

**Published:** 2021-10-06

**Authors:** Sirin Apiyasawat, Sakaorat Kornbongkotmas, Ply Chichareon, Rungroj Krittayaphong, for the COOL‐AF Investigators

**Affiliations:** ^1^ Faculty of Medicine, Ramathibodi Hospital Mahidol University Bangkok Thailand; ^2^ Queen Savang Vadhana Memorial Hospital Chonburi Thailand; ^3^ Faculty of Medicine Prince of Songkla University Songkla Thailand; ^4^ Faculty of Medicine Siriraj Hospital Mahidol University Bangkok Thailand

**Keywords:** atrial fibrillation, atrial fibrillation type, mortality, registry, stroke

## Abstract

**Aims:**

Persistent and permanent atrial fibrillation (AF) often occurs in the presence of multiple comorbidities and is linked to adverse outcomes. It is unclear whether the sustained pattern of AF itself is prognostic or if it is confounded by underlying comorbidities. Here, we tested the association between the temporal patterns of AF and the risks of ischemic stroke and all‐cause mortality.

**Methods and Results:**

In a prospective multicenter cohort, 3046 non‐valvular AF patients were consecutively enrolled and followed for adverse outcomes of all‐cause mortality and ischemic stroke. The risks of both outcomes were adjusted for underlying comorbidities, and compared between the patterns of AF. At baseline, the patients were classified as paroxysmal (N = 963, 31.6%), persistent (N = 604, 19.8%), and permanent AF (N = 1479, 45.6%) according to the standard definition. Anticoagulants were administered in 75% of all patients and 83% of those with CHA_2_DS_2_‐VAS_c_ score ≥2 in males or ≥3 in females. During a mean follow up of 26 (SD 10.5) months, all‐cause mortality occurred less in paroxysmal AF (2.5 per 100 patient‐years) than in persistent AF (4.4 per 100 patient‐years; adjusted hazard ratio [HR] 0.66, 95% CI, 0.46‐0.96; *P* = .029) and permanent AF (4.1 per 100 patient‐years; adjusted HR 0.71, 95% CI, 0.52‐0.98; *P* = .036). The risk of ischemic stroke was similar across all patterns of AF.

**Conclusions:**

In this multicenter cohort of AF patients, persistent and permanent AF was associated with higher all‐cause mortality than paroxysmal AF, independent of baseline comorbidities.

**Clinical Trial Registration:**

Thai Clinical Trial Registration; Study ID: TCTR20160113002 (http://www.thaiclinicaltrials.org/show/TCTR20160113002).

## Graphical abstract

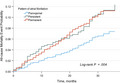

## 1 INTRODUCTION

Atrial fibrillation (AF) is categorized into paroxysmal, persistent, and permanent according to temporal patterns. Studies have been shown that approximately one‐third^1^ to one‐fourth^2^ of patients with paroxysmal AF progress to persistent AF within 10 years. This ratio is even higher in the presence of structural heart disease—reportedly more than half progressing in 10 years.^3^ Those with persistent forms of AF are linked to higher comorbidities and worse clinical outcomes.^2^, ^4^, ^5^, ^6^, ^7^

Current recommendation for management of AF focuses on controlling comorbidities to reduce adverse events rather than interrupting the progression of AF.^8^ However, recent trials suggested that the temporal pattern of AF itself may be an independent predictor of clinical outcomes.^4^, ^9^ Persistent form of AF can lead to both atrial and ventricular remodeling that could subsequently progress to AF‐mediated cardiomyopathy.^10^, ^11^, ^12^ Interrupting the progression of AF at the early stage has also recently been shown to reduce stroke and cardiovascular death.^13^

Here, we aim to investigate the association between the temporal patterns of AF and adverse clinical outcomes in the COOL‐AF registry (**Co** hort of Antithrombotic Use and **O** ptimal INR **L** evel in Patients with Non‐Valvular **A** trial **F** ibrillation in Thailand), a nationwide prospective cohort of AF patients. We hypothesize that paroxysmal AF was associated with lower mortality and stroke risks than persistent or permanent AF.

## 2 METHODS

### 2.1 Study population

COOL‐AF registry is a multicenter prospective cohort study of patients with AF patients without significant valvular diseases. The study consecutively enrolled patients from 27 hospitals^14^ across all regions of Thailand. The inclusion and exclusion criteria were described previously.^14^ Briefly, adults (ie, age >18) with electrocardiography‐confirmed AF were eligible for the enrolment. The exclusion criteria were (i) ischemic stroke within 3 months; (ii) hematologic disorders that can increase the risk of bleeding such as thrombocytopenia (<100 000/mm^3^) and myeloproliferative disorders; (iii) mechanical prosthetic valve or valve repair; (iv) rheumatic valve disease or severe valve disease; (v) AF associated with transient reversible cause; (vi) current participation in a clinical trial; (vii) life expectancy <3 years; (viii) pregnancy; (ix) inability to attend follow‐up visits; and (x) refusal to participate in the study. The protocol was approved by the ethics committee of each participating hospital. All patients provided written informed consent.

### 2.2 Data collection

The source of data were medical records and patients or family interviews. Data collected at baseline included demographic profile, body weight and height, temporal pattern of AF, medical history, clinical examination, laboratory data, medication use, and all components CHA_2_DS_2_‐VAS_c_ and HASBLED scores. In patients implanted with cardiac implantable electronic devices (CIEDs), AF burden was recorded from the device interrogation data. All patients were prospectively followed every 6 months for a total of 36 months. Those with newly diagnosed AF and those with incomplete data were excluded from this analysis. Data collected during follow up included medical history, clinical outcomes, medications, and laboratory data.

The enrolment and all data acquisition occurred between 2014 and 2017. All data were entered in a predefined web‐based form and centrally validated for completion. Monitoring visits were conducted at all participating sites to ensure compliance with the protocol and validity of the data.

### 2.3 Definitions

Temporal patterns of AF were classified by investigators as paroxysmal (AF that terminates spontaneously or with intervention within 7 days of onset), persistent (AF that is continuously sustained beyond 7 days), and permanent (AF that is accepted by the patient and physician, and no further attempts to restore/maintain sinus rhythm will be undertaken.) according to the standard guideline.^8^, ^15^ Congestive heart failure (CHF), coronary artery disease (CAD), and vascular disease were defined using the same criteria of those used in CHA_2_DS_2_‐VAS_c_ score.^8^, ^15^ Briefly, CHF was described as clinical heart failure, or objective evidence of moderate to severe left ventricular dysfunction, or hypertrophic cardiomyopathy, CAD as angiographically significant coronary artery disease or previous myocardial infarction, and vascular disease as the presence of CAD, peripheral artery disease, or presence of aortic plaque. Presence of dialysis, transplant, serum creatinine >200 µmol/L was classified as abnormal renal function, and presence of cirrhosis, bilirubin >×2 upper limit of normal, AST/ALT/ALP >3× upper limit of normal as abnormal liver function, according to the criteria used in HASBLED score.^8^, ^15^ Risk of ischemic stroke was assessed by CHA_2_DS_2_‐VAS_c_ score and further classified as low (CHA_2_DS_2_‐VAS_c_ score = 0 in males, or 1 in females), intermediate (CHA_2_DS_2_‐VAS_c_ score of 1 in males or 2 in females), and high (CHA_2_DS_2_‐VAS_c_ score ≥2 in males or ≥3 in females), as recommended by the standard guideline.^8^

### 2.4 Assessment of clinical outcomes

Patients were prospectively followed for the primary outcomes of all‐cause mortality and ischemic stroke. The secondary outcomes were cardiovascular death, major bleeding, and intracranial hemorrhage. The occurrence of these events was monitored every 6 months until the end of follow‐up at 36 months. All events were validated by the adjudication committee. Ischemic stroke was defined as an episode of neurological dysfunction caused by focal cerebral, spinal, or retinal infarction.^16^ Major bleeding was defined according to the International Society of Thrombosis and Haemostasis criteria,^17^ which were fatal bleeding, bleeding in a critical area or organ(s), bleeding that results in a decrease in hemoglobin level of at least 20 g/L, and bleeding that requires a transfusion of at least 2 units of red cells.

### 2.5 Statistical analysis

Continuous data were reported as mean (standard deviation, SD); categorical data, as number (percentage). The event rates of the clinical outcomes were measured as patient‐year. Differences in baseline characteristics between the patterns of AF were compared by using one‐way analysis of variance with post hoc least significant difference test for continuous variables and Chi‐square test with post hoc pairwise comparisons for categorical variables. Kaplan‐Meier with log‐rank test was used to estimate the survival differences between the patterns. Hazard ratios (HRs) with 95% confidence intervals (CI) of clinical outcomes were calculated by Cox proportional hazards models. Using backward step‐wise selection method, the models were adjusted with age, gender, body mass index, smoking status, alcohol use, hypertension, dyslipidemia, bleeding history, CHF, diabetes mellitus, history of stroke or transient ischemic attack (Stroke/TIA), vascular disease, abnormal renal function, abnormal liver function, use of antiplatelet, and use of anticoagulant. Validation for proportional hazards assumption was performed using Schoenfeld tests for all global models and scaled Schoenfeld residuals for each covariate.^18^ There were no violations detected graphically or by Grambsch‐Therneau tests (ie, all *P* values for global models and for each covariate were >0.05). Statistical adjustment for multiplicity was not performed because of the exploratory nature of this trial.^19^ A *P* value of <0.05 was considered significant. All analyses were conducted using IBM SPSS Statistics for Windows, Version 25.0 (IBM Corp.) and Stata Statistical Software: Release 13 (StataCorp LP).

## 3 RESULTS

### 3.1 Baseline findings

**FIGURE 1:**
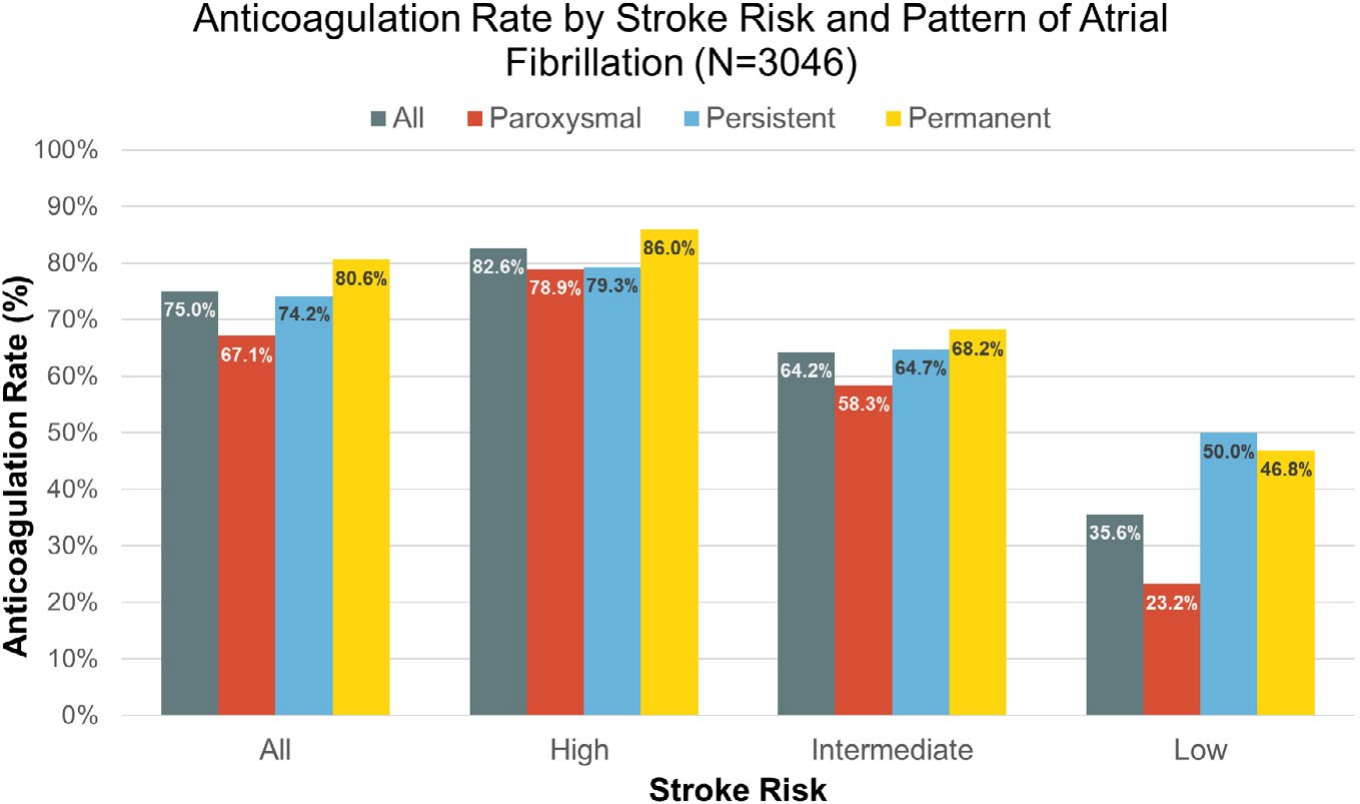
Anticoagulation rate by stroke risk and pattern of atrial fibrillation. Stroke risks were categorized as low (CHA_2_DS_2_‐VAS_c_ score =0 in males, or 1 in female), intermediate (CHA_2_DS_2_‐VAS_c_ score of 1 in males or 2 in females), and high (CHA_2_DS_2_‐VAS_c_ score ≥2 in males or ≥3 in females)

A total of 3402 patients were enrolled in the COOL‐AF registry. After excluding the patients with newly diagnosed AF (N = 91) and those with incomplete data (N = 275), 3046 patients (mean age 67.1, 41% female, mean CHA_2_DS_2_‐VAS_c_ score 2.97) remained in the analysis. Of those, 963 patients (31.6%) were classified as paroxysmal AF, 604 patients (19.8%) as persistent AF, and 1479 patients (48.6%) as permanent AF (Table 1). Patients with paroxysmal AF were more likely female, less likely to have history of CHF, and had lower CHA_2_DS_2_‐VAS_c_ and HASBLED scores than those with persistent and permanent AF. The mean age of those with paroxysmal (65.9 years) and persistent (66.8 years) AF were relatively similar (*P* = .105 between paroxysmal and persistent), but younger than those with permanent AF (mean age 68.1, *P* < .001 vs paroxysmal and = 0.019 vs persistent). Risk of ischemic stroke was classified as high (CHA_2_DS_2_‐VAS_c_ score ≥2 in males or ≥3 in females) in 2239 patients (73.5%). The overall anticoagulation rate in this group (Figure 1) was 82.6% (N = 1849), highest in permanent AF (N = 985, 86.0%), followed by persistent (N = 349, 79.3%, *P* vs permanent <0.001) and paroxysmal (N = 515, 78.9%, *P* vs permanent = 0.001). Of all anticoagulants prescribed (N = 2286), vitamin K antagonist (VKA) was the most frequently used (N = 2103, 92%). A total of 75 (2.5%) and 92 (3%) patients underwent cardioversion and catheter ablation for AF respectively (Table S1). CIEDs were implanted in 282 patients (9.3%). Of those, AF burden was recorded in 92 patients with a mean of 34.4% (SD 40.1) (Table S1).

**TABLE 1:** Baseline characteristics by patterns of atrial fibrillation

	Px (N = 963)	Ps (N = 604)	Pm (N = 1479)	Pair‐wise *P* value		
				Px vs Ps	Px vs Pm	Ps vs Pm
Demographics						
Age, mean (SD), y	65.9 (11.3)	66.8 (11.1)	68.1 (11.0)	0.105	<0.001	0.019
Female, No. (%)	461 (47.9%)	221 (36.6%)	578 (39.1%)	<0.001	<0.001	0.862
BMI, mean (SD), m^2^/kg	25.2 (4.7)	25.5 (4.7)	25.1 (4.8)	0.297	0.654	0.132
Medical history						
Current smoker, No. (%)	20 (2.1%)	20 (3.3%)	58 (3.9%)	0.132	0.011	0.506
Alcohol use, No. (%)	31 (3.2%)	26 (4.3%)	71 (4.8%)	0.264	0.056	0.626
Hypertension, No. (%)	642 (66.7%)	409 (67.7%)	1032 (69.8%)	0.667	0.106	0.355
Diabetes, No. (%)	242 (25.1%)	147 (24.3%)	368 (24.9%)	0.724	0.890	0.794
CAD, No. (%)	162 (16.8%)	116 (19.2%)	202 (13.7%)	0.229	0.032	0.001
Stroke/TIA, No. (%)	141 (14.6%)	94 (15.6%)	284 (19.2%)	0.619	0.004	0.051
Vascular disease, No. (%)	168 (17.4%)	125 (20.7%)	215 (14.5%)	0.108	0.053	0.001
CHF, No. (%)	154 (15.9%)	172 (28.5%)	404 (27.3%)	<0.001	<0.001	0.591
Abnormal renal function, N (%)	30 (3.1%)	21 (3.5%)	44 (3.0%)	0.695	0.843	0.550
Abnormal liver function, No. (%)	22 (2.3%)	16 (2.6%)	33 (2.2%)	0.648	0.931	0.568
Dementia, No. (%)	6 (0.6%)	4 (0.7%)	14 (0.9%)	0.924	0.386	0.525
Bleeding history, No. (%)	88 (9.1%)	52 (8.6%)	159 (10.8%)	0.721	0.197	0.142
Medications						
Antiplatelet, No. (%)	289 (30.0%)	188 (31.1%)	332 (22.4%)	0.640	<0.001	<0.001
OAC, No. (%)	646 (67.1%)	448 (74.2%)	1192 (80.6%)	0.003	<0.001	0.001
VKA, No. (%)	557 (57.8%)	415 (68.7%)	1131 (76.5%)	0.007	<0.001	0.182
DOACs, No. (%)	89 (9.2%)	33 (5.5%)	61 (4.1%)	0.007	<0.001	0.182
Antiplatelet and OAC, No. (%)	101 (10.5%)	70 (11.6%)	104 (7.0%)	0.885	<0.001	<0.001
Risk scores						
CHA_2_DS_2_‐VAS_c_, mean (SD)	2.82 (1.7)	2.95 (1.6)	3.08 (1.6)	0.119	<0.001	0.102
HASBLED, mean (SD)	1.44 (1.0)	1.51 (1.0)	1.61 (1.0)	0.140	<0.001	0.042
Stroke risk*, NO. (%)						
Low	142 (14.7%)	48 (7.9%)	94 (6.7%)	<0.001	<0.001	0.191
Intermediate	168 (17.4%)	116 (19.2%)	139 (16.2%)	0.379	0.405	0.093
High	653 (67.8%)	440 (72.8%)	1146 (77.5%)	0.035	<0.001	0.024

Abbreviations: BMI, body mass index; CAD, coronary artery disease; CHF, congestive heart failure; DOAC, direct oral anticoagulant; OAC, oral anticoagulant; Pm, permanent atrial fibrillation; Ps, persistent atrial fibrillation; Px, paroxysmal atrial fibrillation; SD, standard deviation; VKA, vitamin K antagonist.

* Stroke risks were categorized as low (CHA_2_DS_2_‐VAS_c_ score = 0 in males, or 1 in female), intermediate (CHA_2_DS_2_‐VAS_c_ score of 1 in males or 2 in females), and high (CHA_2_DS_2_‐VAS_c_ score ≥2 in males or ≥3 in females).

### 3.2 Outcomes

**FIGURE 2:**
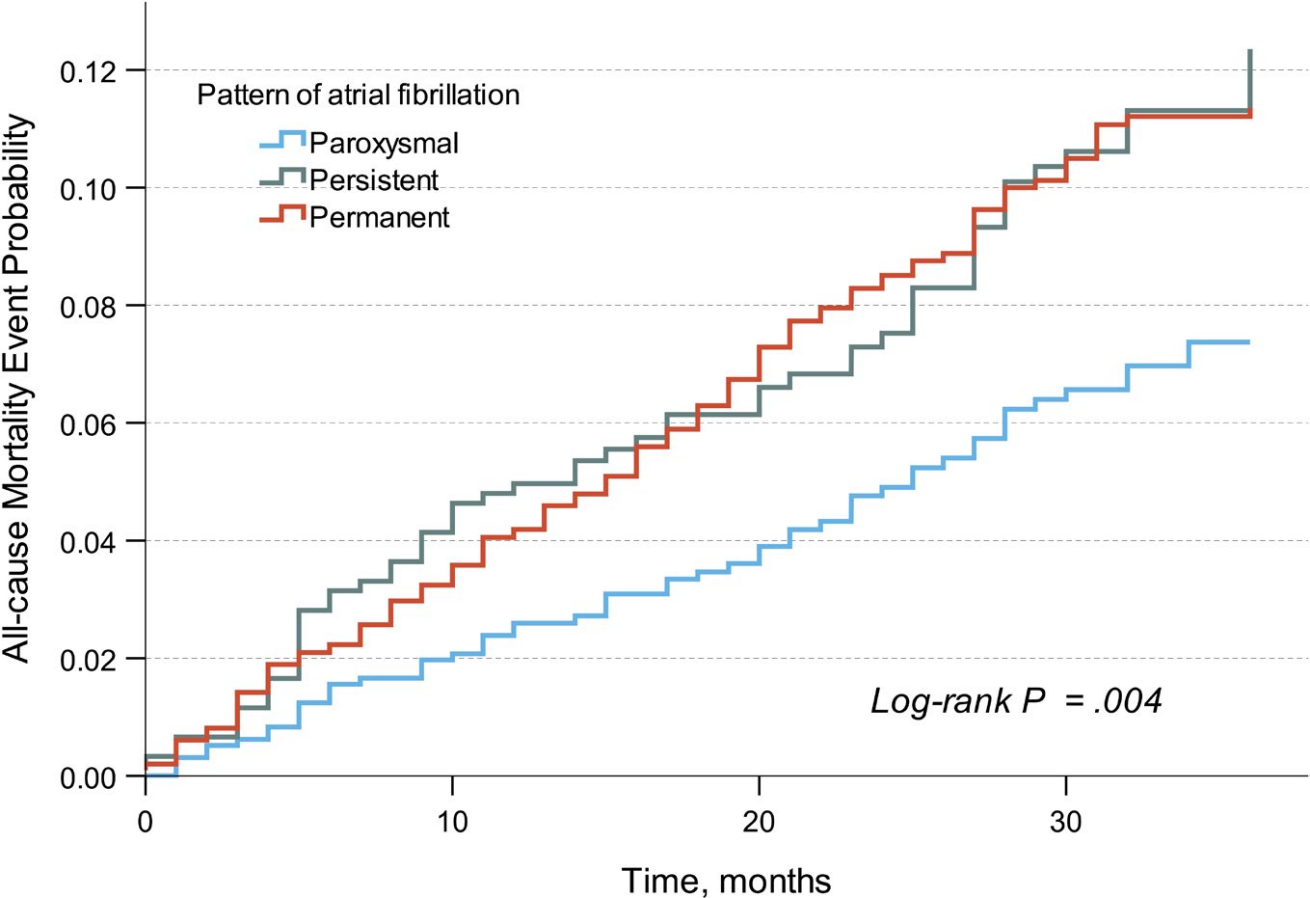
Kaplan‐Meier cumulative probability curve of all‐cause mortality by pattern of atrial fibrillation

**FIGURE 3:**
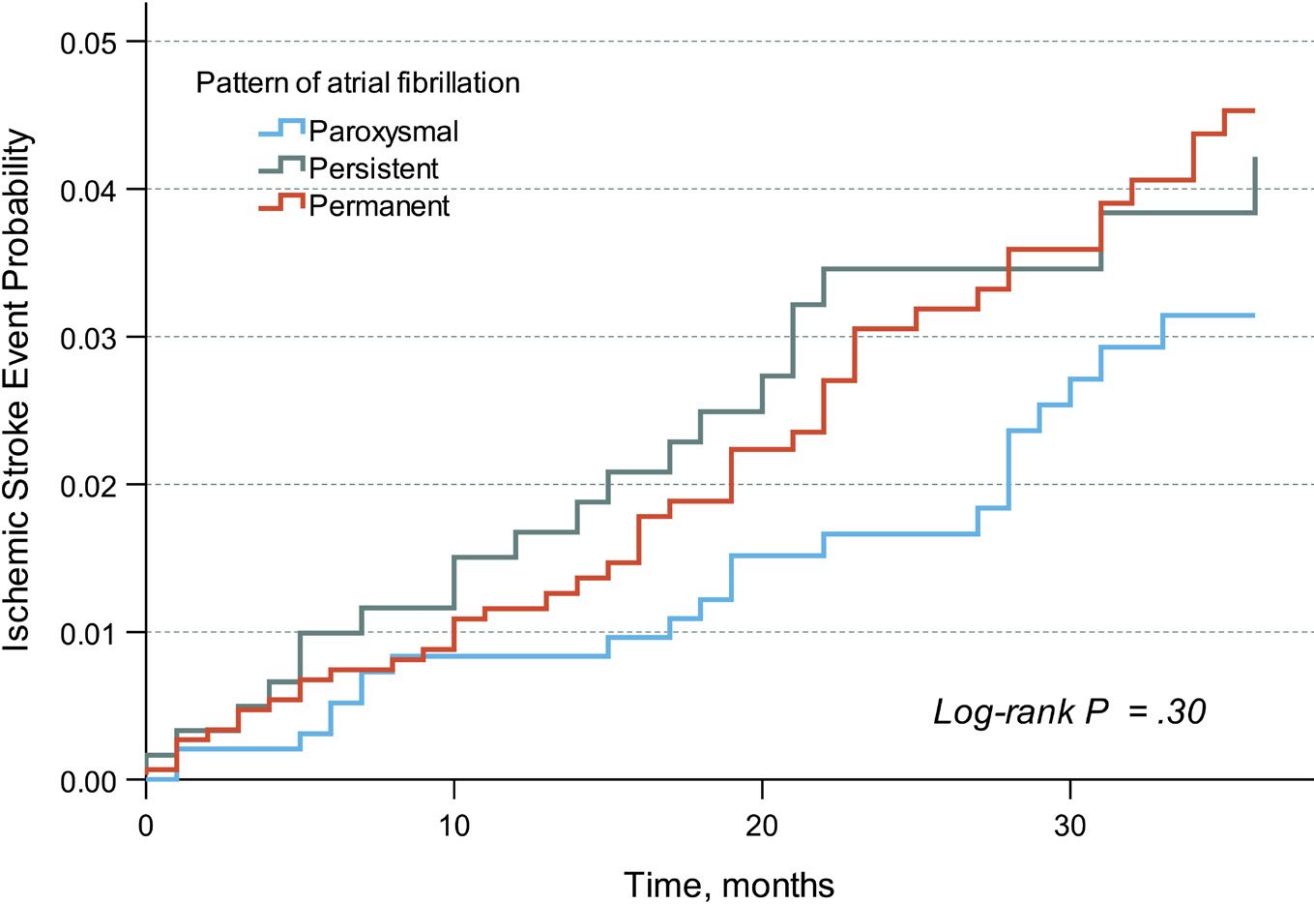
Kaplan‐Meier cumulative probability curve of ischemic stroke by pattern of atrial fibrillation

During a mean follow up of 26 (SD 10.5) months, 240 patients died (7.9%; 3.6 per 100 patient‐years). The causes of death (Table 2) were cardiovascular in 82 patients (34.2%), noncardiovascular in 110 patients (45.8%), and undetermined in 48 patients (20%). Those with paroxysmal AF were less likely to die of any causes (2.5 per 100 patient‐years) than those with persistent AF (4.4 per 100 patient‐years; adjusted HR 0.66, 95% CI, 0.46‐0.96; *P* = .029) and those with permanent AF (4.1 per 100 patient‐years; adjusted HR 0.71, 95% CI, 0.52‐0.98; *P* = .036; Figure 2 and Table 3). There were no differences in the incidence rates of cardiovascular death, noncardiovascular death, ischemic stroke (Figure 3), and intracranial hemorrhage between the patterns of AF (Table 3). Major bleeding was fewer in patients with paroxysmal AF (1.5 per 100 patient‐years) than those with persistent AF (2.6 per 100 patient‐years; adjusted HR 0.59, 95% CI, 0.67‐0.97; *P* = .037; Table 3). Comparing persistent and permanent AF, the incidences of all‐cause mortality, cardiovascular death, ischemic stroke, and intracranial hemorrhage were not statistically different.

**TABLE 2:** Causes of death by pattern of atrial fibrillation

Causes of death	Paroxysmal AF (N = 56)	Persistent AF (N = 59)	Permanent AF (N = 125)	Total deaths (N = 240)
CV death	19 (33.9%)	21 (35.6%)	42 (33.6%)	82 (34.2%)
Heart failure	8	3	9	20
Sudden cardiac death	2	4	7	13
Myocardial infarction	1	4	4	9
Intracerebral hemorrhage	6	3	13	22
Stroke	1	4	9	14
Other CV deaths	1	3	0	4
Non‐CV death	26 (46.4%)	26 (44.1%)	58 (46.4%)	110 (45.8%)
Infection/Sepsis	16	15	30	61
Malignancy	2	3	8	13
Pulmonary	2	2	2	6
Trauma	1	0	3	4
Hemorrhage with neither CV bleeding or stroke	1	2	6	9
Other non‐CV deaths	4	4	9	17
Undetermined	11 (19.6%)	12 (20.3%)	25 (20%)	48 (20%)

Abbreviations: AF, atrial fibrillation; CV, cardiovascular.

**TABLE 3:** Clinical outcomes by pattern of atrial fibrillation

	Incidence (per 100 patient‐years)				Px vs Ps		Px vs Pm		Ps vs Pm	
	All	Px	Ps	Pm	HR* (95% CI)	*P*	HR* (95% CI)	*P*	HR* (95% CI)	*P*
All‐cause mortality	3.6	2.5	4.4	4.1	0.66 (0.46‐0.96)	.029	0.71 (0.52‐0.98)	.036	1.07 (0.78‐1.47)	.655
CV death	1.2	0.9	1.6	1.4	0.62 (0.33‐1.17)	.143	0.70 (0.4‐1.26)	.204	1.12 (0.65‐1.91)	.684
Non‐CV death	1.7	1.2	1.9	1.9	0.66 (0.38‐1.14)	.136	0.68 (0.42‐1.09)	.110	1.03 (0.64‐1.66)	.899
Ischemic stroke	1.3	1.0	1.5	1.4	0.72 (0.39‐1.32)	.286	0.73 (0.43‐1.23)	.235	1.01 (0.59‐1.74)	.956
Major bleeding	2.1	1.5	2.6	2.4	0.59 (0.36‐0.97)	.037	0.71 (0.46‐1.09)	.122	1.20 (0.79‐1.81)	.396
Intracranial hemorrhage	0.7	0.6	0.5	0.8	1.21 (0.49‐3.03)	.679	0.92 (0.47‐1.79)	.813	0.76 (0.33‐1.77)	.527

Abbreviations: CV, cardiovascular; HR, hazard ratio; Pm, permanent atrial fibrillation; Ps, persistent atrial fibrillation; Px, paroxysmal atrial fibrillation.

* HR adjusted for age, gender, body mass index, smoking status, alcohol use, hypertension, dyslipidemia, bleeding history, congestive heart failure, diabetes mellitus, stroke or transient ischemic attack, vascular disease, abnormal renal function, abnormal liver function, use of antiplatelet agent, and use of anticoagulation agents.

## 4 DISCUSSION

In this perspective, multicenter nationwide cohort of moderately well‐anticoagulated AF patients, excess mortality risks were demonstrated in persistent and permanent AF over paroxysmal AF. The increase in mortality risk was independent of age, gender, use of anticoagulant, history of bleeding, and multiple comorbidities. The risks of ischemic stroke, on the other hand, were statistically similar across all patterns of AF.

The prognostic significance of AF pattern has been analyzed in many trials and registries. Among selected, well‐controlled, and well‐anticoagulated population (ENGAGE AF‐TIMI 48,^5^ ROCKET‐AF,^20^ and AMADEUS^21^ trials), nonparoxysmal AF was independently associated with worse survival and higher thrombo‐embolic event than paroxysmal AF. The results have been conflicting in “real‐world” populations. In the Loire valley atrial fibrillation project,^22^ age and comorbidities, rather than a pattern of AF, were associated with risk of stroke and all‐cause mortality. In the Fushimi^9^ and GARFIELD‐AF^23^ registries, the independent association between adverse clinical outcomes and AF pattern were demonstrated but with some dissimilarity. In anticoagulated patients, one registry^9^ showed a prognostic significance of AF pattern in ischemic stroke but not all‐cause mortality while a different study showed the contrary.^23^

Besides geographic area, there were some similarity and dissimilarity between the above‐mentioned registries and ours. We enrolled a higher percentage of nonparoxysmal AF (68%) than the above‐mentioned registries (42% in Loire valley registry^22^ and 51% in GARFIELD‐AF^23^). Similar to all of these registries, VKA was the main anticoagulant. Time in therapeutic range (TTR) in our population, reported elsewhere,^24^ was achieved in 53.6% of 2233 patients taking VKA. Though suboptimal, the number was in line with the TTR of 51.4% in GARFILED‐AF.^25^ The anticoagulation rate, however, was different between our registry and others. In our trial, there were more patients who received anticoagulants (75% of all patients, 83% of patients with high stroke risk) than those in other registries (40% in Loire valley registry,^22^ 53% in Fushimi registry,^9^ and 68% in GARFIELD‐AF^23^). We detected no differences in risks of ischemic stroke across all patterns of AF. This finding could likely be explained by the adequacy of anticoagulation in our trial.

Multiple differences in baseline characteristics were detected between the patterns of AF in our patients. Those with paroxysmal AF were younger and less likely to have a history of CHF than those with nonparoxysmal AF. On the other hand, among patients at high risk for ischemic stroke, those with nonparoxysmal AF were better anticoagulated than those with paroxysmal AF. The imbalances in baseline risk factors, however, were all adjusted in a well‐validated regression model. The regression analysis revealed an approximately 30% increase in mortality risk of nonparoxysmal over paroxysmal AF. Among the nonparoxysmal patterns themselves (persistent and permanent AF), the risks were statistically similar. These results support the concept that nonparoxysmal pattern of AF was not just a term of duration and frequency—rather, it was a disease state—an advanced form associated with worse outcomes.^26^ Finally, the increase in mortality risk was not driven by any particular causes of death either cardiovascular or noncardiovascular. As a matter of fact, noncardiovascular death occurred more frequently than cardiovascular death suggesting that a more comprehensive approach in AF management is essential to improving outcomes in our patients.

### 4.1 Strengths

The study design was prospective and multicenter. Data collection was validated and adjudicated. The incidences of the major outcomes were comparable to those reported globally and regionally^9^, ^22^, ^27^, ^28^, ^29^ reflecting the quality of AF management and the adequacy of event reporting. Finally, unlike most of the clinical trials,^30^ the Cox regression models in our trial were validated for proportional assumptions.

### 4.2 Limitations

As a result of the nature of the registry, only the association, not the causation, could be demonstrated. Baseline characteristics were not balanced and the therapeutic strategy was not controlled between groups. Despite the statistical adjustment, residual confounders remained. One potential confounder was AF burden which was measured only in a small number of patients. Another was the change of AF pattern during the follow up which was not recorded in this trial.

Similar to previous trials, the AF pattern was defined clinically at physician discretion, which is known to be subject to misclassification error.^5^, ^6^, ^9^, ^22^, ^23^ Vitamin K antagonist was the main choice of anticoagulant. The use of direct oral anticoagulant (DOAC) was limited by the reimbursement policy. The follow‐up time was relatively short and may have some effects on the prognosis of chronic disease like AF. Lastly, the cause of death was reported as “undetermined” in 20% of all deaths. However, the prognostic significance of any specific causes of death was not the primary research question here.

## 5 CONCLUSION

In this prospective multicenter cohort of moderately well‐anticoagulated AF patients without significant valvular diseases, the temporal pattern of AF was prognostic for all‐cause mortality but not for ischemic stroke. Nonparoxysmal AF was associated with an approximately 30% increase in all‐cause mortality.

## CONFLICT OF INTEREST

Authors declare no conflict of interests for this article.

## AUTHOR CONTRIBUTION

SA and RK had the idea for and designed the study and were responsible for the overall content as guarantors. All authors collected the data. SA performed the statistical analysis. SA mainly wrote the manuscript with support from SK, PC, and RK. All authors provided critical feedback and contributed to the final manuscript.

## PATIENT AND PUBLIC INVOLVEMENT

Patients or the public were not involved in the design, or conduct, or reporting, or dissemination plans of our research.

## PATIENT CONSENT FOR PUBLICATION

Not required.

## ETHICS APPROVAL

The protocol for this study was approved by the institutional review boards (IRBs) of the Thailand Ministry of Public Health (which covers IRBs for Buddhachinaraj Hospital, Chiangrai Prachanukroh Hospital, Chonburi Hospital, Lampang Hospital, Maharat Nakorn Ratchasima Hospital, Nakornping Hospital, Prapokklao Hospital (Chanthaburi), Ratchaburi Hospital, Surat Thani Hospital, Surin Hospital, Udonthani Hospital, and Sapphasitthiprasong Hospital) and Central Research Ethics Committee (CREC, which covers IRBs for Central Chest Institute of Thailand, Charoen Krung Pracha Rak Hospital, Chiang Mai Hospital, King Chulalongkorn Memorial Hospital, Naresuan University Hospital, Songklanakarind Hospital, Ramathibodi Hospital, Siriraj Hospital, Thammasat Hospital, Golden Jubilee Medical Center, Srinakarind Hospital, Phramongkutklao Hospital, Police General Hospital, and Faculty of Medicine Vajira Hospital) and IRB of Queen Savang Vadhana Memorial Hospital. All patients provided written informed consent prior to participation.

## Supporting information

Table S1

## Data Availability

The individual anonymized data supporting the analyses contained in the manuscript will be made available upon reasonable written request.
