# Supplementary material for: Mortality risk and temporal patterns of atrial fibrillation in the nationwide registry
Source: J Arrhythm. 2021 Oct 6;37(6):1434–42. doi: 10.1002/joa3.12643 (PMC8637082; doi:10.1002/joa3.12643)
Supplement: Supplementary file 1 — Table S1 [file JOA3-37-1434-s001.docx]

# **Table S1.** Additional Baseline Characteristics by Patterns of Atrial Fibrillation.

|  | **Paroxysmal AF (N=963)** | **Persistent AF (N=604)** | **Permanent AF (N=1479)** | **All (N=3046)** |
| --- | --- | --- | --- | --- |
| **History of Catheter Ablation for AF, No. (%)** | **61 (6.3%)** | **16 (2.6%)** | **15 (1.0%)** | **92 (3.0%)** |
| Pulmonary Vein Isolation, No. | 19 | 2 | 5 | 26 |
| CFAE, No. | 40 | 12 | 11 | 63 |
| **History of Cardioversion, No. (%)** | **31 (3.2%)** | **28 (4.6%)** | **16 (1.1%)** | **75 (2.5%)** |
| **CIEDs, No. (%)** | **150 (15.6%)** | **42 (7.0%)** | **90 (6.1%)** | **282 (9.3%)** |
| AF Burden Availability, No. (%) | 67 | 12 | 13 | 92 |
| AF Burden, mean (SD), % | 24.12 (33.3) | 57.15 (46.6) | 66.2 (43.2) | 34.38 (40.1) |

Abbreviations: AF, atrial fibrillation; CIEDs, cardiac implantable electronic devices; CFAE, complex fractionated atrial electrogram; SD, standard deviation.
